# Tuberculosis Contact Screening and Isoniazid Preventive Therapy Among Children Under 5 in the Mbeya and Songwe Regions, Tanzania

**DOI:** 10.24248/EAHRJ-D-17-00186

**Published:** 2018-04-01

**Authors:** Yesaya K Mwasubila, Issa Sabi, Rogatus Kabyemera, Nyanda E Ntinginya, Reginald Sauve, Benson Kidenya

**Affiliations:** a Department of Health, Songea District Council, Ruvuma, Tanzania; b School of Public Health, Catholic University of Health and Allied Sciences, Mwanza, Tanzania; c Mbeya Medical Research Centre, National Institute for Medical Research, Mbeya, Tanzania; d Department of Paediatrics and Child Health, Bugando Medical Centre, Mwanza, Tanzania; e Department of Paediatrics and Child Health, Catholic University of Health and Allied Sciences, Mwanza, Tanzania; f Faculty of Medicine, University of Calgary, Calgary, Alberta, Canada; g Department of Biochemistry, Catholic University of Health and Allied Sciences, Mwanza, Tanzania

## Abstract

**Introduction:**

The World Health Organization (WHO) recommends contact screening and initiation of isoniazid preventive therapy (IPT) for children under 5 years of age exposed to a sputum smear-positive (SS+) tuberculosis (TB) source case. We conducted this study in order to assess implementation of these recommendations in southwestern Tanzania.

**Methods:**

We conducted this cross-sectional study from June to August 2015 in 12 selected health facilities in the Mbeya and Songwe regions of Tanzania. Adult SS+ pulmonary TB patients living in the same household as children under 5 were enrolled. Structured questionnaires were used to obtain sociodemographic information and details about screening and intervention activity related to contact children under 5. Data were analysed using Stata version 11.0.

**Results:**

We enrolled 257 index cases, who collectively had 433 contact children under 5. The median age of the index cases was 34 years (interquartile range 28 to 41) and 52.9% were male. Out of 433 contacts, 31 (7.2%) were screened for TB, of whom 7 (22.6%) were treated for presumptive TB. Among those screened, 24 were not diagnosed with TB, of whom only 8 (33.3%) received IPT.

**Conclusion:**

Low uptake of TB contact screening and IPT administration among eligible children under 5 was observed in this study. Health-care workers should be sensitized to screening of household contacts of adults with SS+ TB and initiate IPT in those who are eligible.

## INTRODUCTION

Childhood tuberculosis (TB) contributes a significant proportion of the global TB burden. In 2015, 10% of the 10.4 million incident TB cases worldwide occurred in children under 15 years of age.^1^ Children are usually infected via household contact with an adult who has sputum smear-positive (SS+) pulmonary TB (PTB).^2^

In line with recommendations set by the World Health Organization (WHO) and governed by national TB programmes, strategies for preventing childhood TB include contact tracing and isoniazid preventive therapy (IPT) for children under 5 in contact with TB source cases.^3,4^ IPT has been shown to reduce the risk of progression from latent TB infection to active TB disease by as much as 59% among children aged 15 years or younger.^5–7^ Despite the known benefits of contact screening and IPT, these strategies remain widely underutilized, even in countries severely affected by TB.^8–12^ For example, a study in Malawi found that only 9% of contacts were screened for TB and only 6% were initiated on IPT.^13^

Tanzania is among the world's 22 high TB-burden countries and childhood TB is estimated to contribute about 10% of the national TB burden.^1^ In keeping with WHO recommendations, Tanzania's National Tuberculosis and Leprosy Control Programme (NTLP) recommends contact investigation and IPT provision for all TB contacts under 5, after excluding active TB disease.^4^ TB treatment services, including IPT initiation and follow-up, are provided for free by the NTLP and supervised by district TB and leprosy coordinators (DTLCs), under the guidance of regional TB and leprosy coordinators (RTLCs).^4^ Despite these recommendations and deployments, uptake of contact tracing and IPT initiation has not been well documented in most facilities, which has made it difficult to evaluate performance and adherence to guidelines. We conducted this study in 2 regions of Tanzania to assess uptake of contact screening and IPT administration, in 2 regions of Tanzania, for children under 5 who are contacts of SS+ TB patients.

## METHODS

### Study Design and Setting

Data for this cross-sectional study were collected from June to August 2015 in the Mbeya and Songwe regions located in the Southern Highlands Zone in southwestern Tanzania. These regions have TB notification rates ranging from 3,700 to 4,000 cases per year, with childhood TB contributing about 10% of the total TB burden. At the time of data collection, the 2 regions had 448 health facilities, which included 18 hospitals, 36 health centres, and 394 dispensaries. We randomly selected 10 hospitals and 2 health centres among sites with TB diagnostic and treatment capabilities.

### Participants

We enrolled consenting adult SS+ PTB patients who lived in the same household with children under 5. Parents or caregivers who had other forms of TB were excluded. A household contact was defined as a child who shared the same house with an index TB case for at least 3 months prior to diagnosis.

### Sample Size and Data Collection

The minimum sample size was estimated using the following formula: n=Z^2^P(1-P)/d^2^, where, Z is the level of confidence that the chosen sample was not representative of the population (using the value 1.96 for 95% confidence interval [CI]), P represents the proportion of the sample that is assumed to practice contact screening (50% or 0.5), and d is margin of error that the probability that the desired sample size was not representative of the study population at 95% CI, expressed as a decimal (0.05). We estimated the minimum sample size to be 384 contact children under 5, but included 433 children in the study to minimize errors. Data were obtained through interviews with adult index patients whose clinic TB treatment cards revealed positive TB results from sputum. Participants confirmed the presence of contact children under 5 before consenting to and proceeding with questionnaire-guided face-to-face interviews during routine TB clinic visits. Interviews captured sociodemographic information and details of contact TB screening, prevention with IPT, and treatment.

### Data Analysis

Completed questionnaires were entered into Microsoft Excel and then exported into Stata version 11.0 (Stata Corp, College Station, TX, USA) for analysis. Categorical variables were summarized as proportions and continuous variables as medians with interquartile range (IQR).

### Ethical Consideration

Ethical approval was obtained from the joint Catholic University of Health and Allied Sciences and Bugando Medical Centre (CUHAS/BMC) Ethics and Review Committee. The respective district administrative authorities granted permission to carry out the study. All patients provided written informed consent before participating.

## RESULTS

### Characteristics of Tuberculosis Index Cases

We enrolled 257 index TB cases, who accounted for 433 contact children under 5. The median age of the index cases was 34 years (IQR 28 to 41) and 136 (52.9%) were male. All index cases had at least 1 contact living in the same household and 150 (58%) were biological parents of the contacts (**Table 1**).

**TABLE 1.** Characteristics of Tuberculosis Index Cases in the Mbeya and Songwe Regions, Tanzania

Characteristic	Frequency (n) or median	% or IQR
**Age, median**	34	28–41
**Sex**		
Male	136	52.9
**Occupation**		
Peasant/subsistence farmer	106	41.3
Pet trader	84	32.7
Housewife	18	7.0
Others	49	19.1
**Education**		
No formal education	23	9.0
Primary education	190	73.9
Secondary education	36	14.0
College education	8	3.1
**Walking time to health facility on foot (minutes)**		
0 to 30	60	23.4
31 to 60	66	25.7
61 to 90	52	20.2
>90	79	30.7
**Number of contacts per household**		
1	125	48.7
2	98	38.1
3	25	9.7
4	9	3.4
**Relation to contact children**		
Mother or father	150	58.4
Guardian/caregiver	107	41.6

Abbreviations: IQR, interquartile range.

### Household Contacts and Isoniazid Preventive Therapy

**FIGURE 1.**
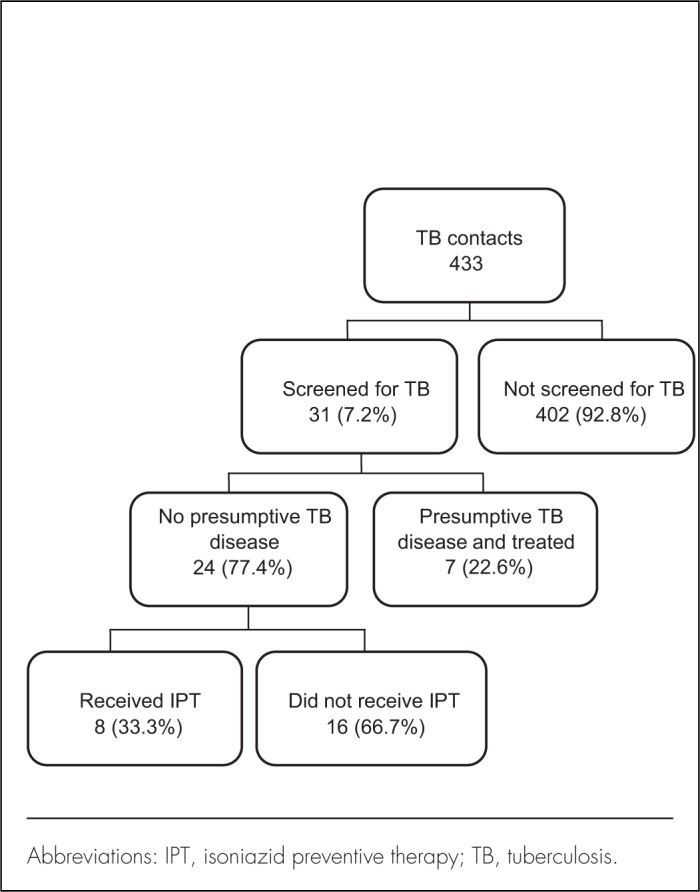
Contact Screening and Isoniazid Preventive Therapy Initiation among Tuberculosis Contact Children Under 5 in the Mbeya and Songwe Regions, Tanzania

Abbreviations: IPT, isoniazid preventive therapy; TB, tuberculosis.

Out of 433 contacts, only 31 (7.2%) were reported to have been screened for TB. Among the 31 who were screened, 7 (22.6%) were found to have presumptive TB disease and were treated with anti-TB therapy. Eight of the 24 remaining contacts (33.3%) started IPT, while the other 16 eligible contacts did not receive any intervention (**Figure 1**).

## DISCUSSION

This study investigated the extent of TB contact investigation as an entry point to IPT initiation among children exposed to adult SS+ PTB patients in southwestern Tanzania. The main purposes of contact investigation were to identify contacts with presumptive TB disease who require anti-TB therapy and to provide a gateway to IPT for those without presumptive TB but who are susceptible to developing disease following recent infection.^7^

Despite the availability of both WHO and national guidelines, which clearly recommend clinical evaluation of household contacts of SS+ PTB adult index cases, only 31 (7.2%) contact children under 5 of SS+ TB source cases were screened for TB. Our findings are similar to screening rates of 9.0% and 5.5% among TB contact children under 5 in Malawi and Vietnam, respectively.^13,14^ The low TB screening rate in our study may have left many children without appropriate interventions, putting them at risk of developing active TB disease.

One-third of the IPT-eligible children – those who were screened and found not to have presumptive TB – were started on therapy. This is lower than proportions reported from studies conducted in Ethiopia (64.3%)^15^ and South Africa (68.0%),^16^ but higher than what has been observed in Malawi (6.0%).^13^ Possible reasons for low IPT uptake in the study area included the limited availability of documentation tools during the study period, which negatively affected the national TB programme's ability to monitor IPT implementation. Contact screening and IPT provision have substantially improved in countries that have introduced IPT registers. For example, in South Africa, the introduction of official documentation forms increased the number of contacts screened per TB patient as well as the proportion of children initiated on IPT.^11^ In India, the introduction of an IPT register increased IPT provision from 19.0% to 61.0%.^17^ As with the low uptake of contact screening, failure to provide IPT to high proportions – two-thirds in our study – of eligible children may contribute to TB-related morbidity and mortality. In South Africa's Western Cape, for example, a study found that 81.4% of missed opportunities for IPT in at-risk children under 3 years of age who later presented with confirmed TB – 25.0% had disseminated TB and 5.1% died.^18^

### Limitations

This study relied solely on patient recall regarding information about contact tracing, TB screening, and IPT administration. Review of IPT provision data tools may have provided a cross-checking mechanism, but unfortunately, these tools were not available in the study sites during the study period. However, this study has provided important evidence to the Tanzanian NTLP on the low uptake of contact investigation and low IPT provision to eligible TB contact children under 5.

## CONCLUSION

The uptake of contact screening and IPT administration among children under 5 observed in this study was low. We advise the NTLP to fully implement the IPT programme and sensitize health-care workers on the importance of contact screening and IPT for those who are eligible. Further research is needed to inform the national TB programme on factors associated with low uptake of contact investigation by health-care workers and the feasibility of implementation of IPT recording tools under the programme setting.
